# Mr. Cross's Sketches of the Medical Schools of Paris

**Published:** 1816-04

**Authors:** 


### *Mr. Cross's Sketches of the Medical Schools of Paris.*

( Continued from p. 262. )

*Aneurism.* The notions which we had entertained of the inferiority of French practice in the treatment of aneurism, are amply confirmed by the testimony of Mr. Cross. Hunter's method of operating at a distance from the seat of the disease, is occasionally tried as an experiment, not adopted as a rule of practice. And even when the surgeon ventures to tie the artery at a distance from the sac, a piece of lint is interposed between the ligatures and the

---

vessel; ligatures of reserve are left loose; and the wound is crammed with lint, to prevent immediate reunion. On heresies like these, we shall make no comment. The admirable treatise of Dr. Jones is unknown, or very imperfectly appreciated, in France. In a case of aneurism at the bend of the arm, consequent on external violence, the operation was witnessed by Mr. Cross. Compression had failed. The subject was a girl of about seven years. After some aukward dissection and groping for the artery with his finger, the surgeon passed a needle with double ligature between the vessel and *biceps muscle*, about an inch above the tumor, and brought it out between the vessel and median nerve. By using the ligature as a hook, the artery was dissected a little from the surrounding parts. A double flat ligature, the eighth of an inch broad, was then passed, by means of a probe, behind the vessel. Of these four ligatures, two were tied at the distance of half an inch from each other; the other two withdrawn. The superior of the two, left on the artery, was a flat one; the inferior was one of the ligatures first passed. The edges of the wound were drawn together by two strips of plaster, encircling the limb and crossing each other posteriorly. The ends of the ligatures were brought out between. A compress and bandage completed the dressing. The weather was then cold; but the child was taken home, and neither seen nor heard of till the morning of the fifth day. She had suffered great pain during the first twenty-four hours; little since. The bandages were removed; but not the plasters, although, in consequence of the tension which had occurred, they had evidently become too tight. Lint, spread with cerate, was applied. The aneurism was then much diminished; the temperature of the limb natural, but no pulsation could be felt in the radial artery. On the ninth day, the ligatures separated; and the wound healed favourably. Richerand, in a case of femoral aneurism, passed four ligatures round the artery, midway between the tumor and the groin. One only was tied, with the intervention of a piece of lint; the remaining three were left loose. The wound was completely stuffed with lint. The ligature had not been tied sufficiently tight: the tumor pulsated slightly. The dressings were removed on the third day. There was pulsation both in the tumor and in the artery, at the point of ligature. Richerand drew the waxed ends of one of the ligatures of reserve through the holes of a brass instrument, called the *presse-artere*; tightened the instrument upon the artery till all pulsation in the tumor had ceased, and there secured it* The two unemployed ligatures of reserve were still left; the wound again crammed with lint; the knee slightly bent; and the limb below surrounded with bags of oat-chaff. The result of this mis-managed case is not known. Temporary compression of the artery, by means of a ligature and the *presse-artere*, has twice been employed in aneurism successfully, by Dubois. Both the instrument and ligature were removed as soon as it was ascertained that on completely loosening the latter, no pulsation of the tumor recurred. "The third case," in which this operation was performed, "has a melancholy and instructive history attached to it. He (Dubois) did not begin to tighten the ligature until the fifth day, on account of hæmorrhage from a wounded femoral vein. On the tenth day, when there was no evident pulsation in the tumor on relaxing the ligature, and when its removal was determined on, hæmorrhage took place from the artery where the ligature and *presse-artere* had been applied, and the patient died during, or very soon after, the amputation of the limb."

*Diseased Testis.*—Castration is considered in no very important light by the French surgeons. "It is bad enough to lose a testicle, but as to the operation, that's nothing," exclaimed M. Roux, to a man on whom he was about to operate. The patient had, some months previously, been cured of hydrocele, by injection of red wine. Enlargement of the testicle remained. It became, at length, very bulky; was sometimes extremely painful. The spermatic cord was not swollen: pressure on its course within the abdomen induced no pain. The organ was examined by candle-light upon a bed, round which dark curtains were closely drawn: the tumor, transparent only at the inferior part, was pronounced to be *hydro-sarcocele*. No palliative plan was recommended. The testicle was removed without consultation or preparatory treatment. The incision of the integuments was made in the usual manner without detaching any portion. Ligatures were immediately applied to both orifices of a divided superficial vessel, and cut off close. The whole of the testis was dissected out ere the cord was divided; its arteries were taken up by a

---

Mr. Cross has given an engraving of this instrument. It is very like the *presse-artere* employed by Mr. Crampton, of Dublin, in his late celebrated operation for popliteal aneurism. The silver forceps of Professor Assalini, are, in Mr. Cross's opinion, preferable to this instrument for exercising compression on an artery.

pair of forceps and tied separately. The ligatures upon these were left of considerable length, and wrapped in linen to prevent entanglement with the dressings. Lint was liberally stuffed into the wound and heaped upon it; and over this five or six linen compresses were secured by a roller. Four days passed before the wound was dressed: it was very large at the end of a week. On the tenth day, all the ligatures were detached. On the fourteenth, the sore had a bad aspect. Pus had formed beneath the skin of the opposite testicle. It was evacuated by an extensive incision, the cavity laid into the general wound, and filled with lint. The cure proceeded very favourably. It seems that M. Roux, on his return from London, instituted a trial of the comparative merits of stuffing the wound with lint, and encouraging union by the first intention in two cases of castration. The result of this experiment determined him to adhere to the former method. We cannot quit this subject without earnestly protesting against the rude and barbarous practice, yet adopted by certain surgeons in this island, of including the whole spermatic cord in the ligature employed on the removal of a morbid testicle.

*Cancer.*—The cancerous mamma is removed by the French in the earliest stage of the disease. They even hesitate not to operate where ulceration has been long established. An example of this kind fell under the observation of Mr. Cross. The patient, aged 38, had, eighteen months before, submitted to removal of a portion of the right breast, affected with open cancer. The disease recurred with extensive ulceration: the axillary glands were enlarged. The operation was again proposed, and the undaunted woman did not reject it. After the tumor had been extirpated, an incision was made from the wound to the centre of the axilla: the diseased glands were sought for and torn away by a silver hook with two prongs. The vessels secured, "four pieces of lint, rolled into masses as big as walnuts, were put into the bottom of the axillary pit, whence the enlarged glands had been dug out." Upon the surface of the large circular wound soft linen was applied; then alternate strata of lint and linen compresses, and the whole secured by a roller. The wound was again dressed on the fourth day; its surface was dark and sloughy; its edges sharp and dry; the discharge fœtid. The stomach of the unfortunate woman could retain no solid food; her extremities were cold, and covered with perspiration; pulse almost imperceptible. She died on the ninth day. The wound had been dressed with common cerate. Immediate union of the wound after removal of the breast has never been attempted till within the last year, and the practice is not yet generally adopted. The arsenical paste* and actual cautery are favourite remedies with the French surgeons, in cancerous affections. The former of these has been applied to the nostrils, and even the mouth and tongue, care being taken that none of it was swallowed, with success. M. Roux explains the non-absorption of the poison into the system, by supposing, that " a state of erethismus" prevails in the lymphatics, Yet deadly effects do occasionally result from its employment. This very writer mentions the case of a patient, who, on the day after the application of the caustic paste, complained of colic and severe vomiting; and in two days perished in convulsions and extreme agony.

" The body went quickly into putrefaction. The internal coat of the stomach, and a great part of the intestinal canal, were inflamed, and marked here and there with dark spots."

Mr. Cross has seen precisely similar effects produced by the " red powder" of some " murdering empiric, applied to incipient cancer of the breast, in London. The pain and inflammation, induced by the action of the arsenical paste, are dreadful. A case, observed by Mr. Cross, will best illustrate the employment of actual cautery in cancer. An aged man had cancer of the lip. The jaw-bone was contaminated. The whole lip was removed by an operation. After three weeks, the wound was covered by ill-looking granulations, and poured out a fœtid discharge. Actual cautery was prefered, in consultation, to the arsenical paste.

" The students surrounded the patient, who was placed on a chair in the middle of the ward, and near him was a red hot cauldron, with irons of different forms, square, round, triangular, and diamond-shaped. M. Pelletan, after brandishing one of these fiery weapons in the air, applied it to the cancerous surface, and kept it there several seconds. He applied four or five of these red hot irons of different forms, till the whole surface was an eschar. Two days afterwards, the eschar had separated; the whole mental portion of the lower jaw was bare; the mental foramen on one side was exposed; and at the centre of the chin the bone was black."

* The French formula for this is seventy parts of cinnabar; twenty-two of sanguis draconis; and eight of oxide of arsenic. This powder is formed with saliva, at the time of application.

Mr. Cross did not remain long enough to witness the issue of the case. In one instance, where a large fungus had arisen in the antrum of the right maxillary bone, he saw the actual cautery applied with excellent effect.

"The tumor was removed through the mouth, by separating the cheek from it on the inside, and a portion of the diseased malar and maxillary bones taken away."

Those parts of the fungus, inaccessible to the knife, were destroyed by the hot iron. The operation was followed by inflammation of the eye and slight eversion of the lower palpebra. The patient was cured in two months, with little deformity.

*Syphilis.*—Corrosive sublimate (muriate of quicksilver) is almost universally employed in the cure of this disease, and apparently with great success. Mr. Cross saw in *L'Hopital des Veneriens,* containing between five and six hundred patients, but very few cases of eruption; none of badly-diseased facial bones; none of destruction of the palate, or sloughing penis. In two or three bad cases only were mercurial friction had recourse to. There were three formulæ of potions in the hospital; a solution of sublimate, decoction of the woods, and a "bland mucilaginous drink." One of these was invariably administered to every patient. Fifty or sixty grains of the muriate are deemed a sufficient dose to effect a cure in confirmed cases; eighteen grains, in common primary sores. A sixteenth or eighth part of a grain is usually given at first, and increased to half a grain. This is, in general, taken at one daily dose. But where the circumstances of the patient will require it to be administered in divided doses, half that quantity is prescribed, evening and morning, in the form either of solution or pills. The dose of muriate was lessened, or altogether omitted, as soon as the mouth became affected. None of the patients suffered from "swollen chops and salivation." Lint only was applied to chancres. Sores on the *labia pudendi* were, in one instance, touched with butter (muriate) of antimony. Buboes were, in general, opened with caustic.

"I know," says Mr. Cross, "but two facts of any value that I ascertained at *L'Hopital des Veneriens,*—the almost universal employment of corrosive sublimate, and the small proportion of secondary and very severe cases of syphilis. The former is a fact not extensively known amongst us; and the latter, few, perhaps, will be prepared to believe."

He thinks that the efficacy of sublimate is undervalued in England; and again, that the syphilis of the French may be so modified by some circumstances, independently of climate, as to yield to treatment comparatively mild. The infected disclose their situation with little reluctance, and the malady is combated at its origin. The common women also "are licensed and registered; medical officers are appointed and salaried by the police for examining them, and reporting the state of their health; and although this system considers public health, perhaps, rather than public morals, if vigorously pursued, it must contribute greatly to the prevention of bad and neglected cases, and to diminish the spreading of venereal infection." And he adds, that on his return to London, he found as many cases of venereal eruption amid the hundred patients at the Lock Hospital, as he had seen in the crowded wards of *L'Hopital des Veneriens*, of Paris. The French surgeons seem to think that lues may arise from gonorrhœa; that the poison of both, therefore, is identically the same. They exercise little patience, or indeed, evince the most reprehensible negligence and precipitation, in deciding upon suspected cases of syphilis. Their opinion frequently rests upon the removal of the disease by mercury. This, we hardly need observe, can constitute no absolute criterion. Dubois pronounced a crustaceous eruption on the forehead of an infant to be venereal, "without asking a question, or examining the child at a nearer distance than three yards." The disease, by Mr. Cross' description, had quite a different character. The woman declared that both she and her husband were free from the contagion.—An elderly man complained of sore throat. Six months previously, he had been cured of a small excrescence on the penis. The surgeon, without inspecting throat or penis, "*jocosely*" denounced the malady as syphilitic, and prescribed the muriate of quicksilver. Upon conduct like this, no animadversion can be sufficiently severe.

*Cutaneous Affections.* In the hospital treatment of *tinea*, the scabs are removed by emollient applications; the head shaved every two or three days, and dressed with an ointment of hydro-sulphuret of potass, in those parts only where the eruption existed. A different method was sometimes employed: poultices having been previously directed, a composition of pure potass and lard, or oil, was used to the scalp, wherever affected by tinea. This caused the hair soon to fall off, or rendered its detachment easy and painless. When the cure was perfected, the hair grew plentifully again on these patches. The disease, if obstinate, is rarely removed in less than six months. Sulphur

Baths or ointment are universally employed for the cure of *itch.* The former is prepared by dissolving four or five ounces of sulphuret of potass in twenty gallons of water, of the temperature of 97° of Fahr. It is to be used once a day for a week or more; but its efficacy is much increased by employing it twice a day. In smell equally offensive, the bath is less speedy in operation than the sulphur ointment. The *liniment savonneux hydro-sulphuré** is a more agreeable application, but inferior in power to the ointment. It must be used twice a day for about a week. An eruption on the hands, arms, and face of a female, denominated *dartre phlyctenoïde* by Alibert, came under the observation of Mr. Cross, in *L'Hopital St. Louis.* There were, in some parts, "simply vesicles; in others, large irregular crusts, the skin around the margin of which was of a dull red colour: their formation was attended by a distressing burning sensation." Quicksilver ointment was applied to one hand; sulphur ointment to the arms; and Van Swieten's liquor (a solution of sublimate) given internally. Salivation came suddenly on, and the mercurial remedies were discontinued.† A case of unique and anomalous disease was also pointed out by Alibert. Each leg, some few inches above the ankle, was surrounded by a zone of preternaturally thickened integument, elevated about the fourth of an inch above the surrounding surface; varying in width from three inches to one; its margins irregular and abrupt; there was neither pain nor soreness.

"The colour of this part was very little deeper than that of the natural skin, and the pores were very much developed and enlarged.—The surface looked so much like the natural skin viewed through a magnifying glass, that I could only call it a growth of the skin to four or five times its usual thickness."

The patient was well; the affection unproductive of inconvenience. Mr. Cross thought, that compression by

---

* R. Saponis albi oz. iv; olei amygdalæ oz. viij; potassæ sulphureti dr. vj; olei thymi guttas xv, vel scrup. j. Dissolve the soap by a water-bath, and mix it with the oil. Then gradually combine the sulphuret, previously powdered or liquified by exposure to the atmosphere.

† Sulphur, in fumigation (or, in other words, the fumes of sulphureous acid), is much employed in dartrous affections, by Alibert, at L'Hopital St. Louis. The application to the surface of the body is partial or general. In the latter case, the patient is receixed into a machine which embraces the neck; but this Mr. Cross was not *permitted* to see. So much for the *philosophical* and *philanthropic* Mons. Alibert.

bandage would have formed a rational mode of treatment. Nothing but poultices was applied. Vaccination seems nearly to have exterminated *the small-pox* in Paris. *L'Hopital des Enfans Malades* receives all the children infected with that disease from the poorer classes. Mr. Cross found, upon his visit there, that no fresh patient had been received in the girls' ward during the last three weeks; and only two in the boys' during the last month. Can the vaccine institutions of our own metropolis make a similar boast?

*Infantile Diseases.* Under the term *gibbosité*, both caries and rickety affections of the spine are included by the French surgeons. Some of their writers have marked the distinction between them. In a rickety child, under three years, with distorted spine, issues had been made on each side of the dorsal vertebræ. Caries, against which only a purulent drain could be usefully instituted, rarely occurs at so young an age. Hence the mischief of confounding, under one title, affections essentially dissimilar, and requiring modes of treatment widely different from each other. Is "the sign of the curvature lateral or forward to be relied on, as indicating whether the deformity be from rickets or caries?" We suspect not invariably. But this is a question more readily proposed than settled. At *L'Hospice de la Maternité* many children have been destroyed by a disease rarely occurring except in hospitals for young subjects *(l'endurcissement du tissu cellulaire—induration of the cellular structure).* About one in twenty suffers from it. The extremities, neck, face, or abdomen, are most commonly attacked.

"The parts affected become swollen, hard, cold to the touch, and of a dull red or livid colour; not pitting from, nor yielding to, pressure with the finger. When the lower extremities are affected, the feet become convex. On dissection, the lymphatic, and generally the mesenteric glands are found much enlarged, and the cellular substance is distended with a yellow serous fluid that is coagulable by boiling water. This disease, when it affects the cheeks, has many symptoms in common with trismus; but I cannot agree to the remark Dr. Jos. Frank made at the Foundling Hospital of Paris, that it has any very great resemblance to tetanus. The appearance, the attitude of the body, the sensations given by the surface of the body to the touch, are different."

In tetanus, no unnatural state of the internal organs is, in general, discovered.

Little remains for us to say in this *concluding section.* Of two of the most splendid improvements of British surgery, the surgical treatment of aneurism, and immediate closure of the wound after various operations with the knife, we have seen that the French very imperfectly appreciate the value, or comprehend the application. And what but the most inflexible pride, and blindest prejudice, can render the minds of the French surgeons insensible to such evidence as is adduced by M. Percy in favour of immediate union after amputation:

"*Ninety-two* patients suffered amputation of the thigh, leg, or arm, by the same hand, on the *22d of September*, in the affair at Nurembourg, and *eighty-six* of them were cured by the *18th of the following month.*"

Yet in the very Memoir upon which this important fact is announced as a comment, Roux declares himself "far from thinking that immediate re-union should be adopted as general practice, or that it is indiscriminately applicable to all cases." With respect to the employment of the *adhesive strap*, so fortunately ushered into notice by Mr. Baynton, they display equal inexperience and prejudice.

"An English surgeon," says Richerand, in his *Nosographie Chirurgicale*, the elementary work on surgery generally read by students in France, "has successfully used adhesive plaster, in the view of approximating the skin over an ulcerated surface. I have employed the same remedy, and invariably observed that cicatrization was, in consequence, much accelerated. *But is such a termination of the disease always desirable? Can the cure of every species of ulcer be attempted without danger?*"

This, with proper deference to the Parisian surgeon, we deem to be a very silly question. The adhesive strap, as a mere remedy—a mean whereby certain objects may be accomplished, can have nothing to do with the *principle* that directs its application. It is wholly subordinate to the views of the operator; and it will be beneficial in proportion as his judgment, respecting the propriety of employing it, has been correctly formed. Are the knife and the saw to be reprobated because every maimed or morbid limb requires not amputation? Now, possibly, Mons. Richerand, after having decided upon the expediency of removing an arm or leg, might select these instruments, as preferable to all others, for the purpose. We, on the very same principle, recommend, against all external wounds and ulcers, *which it is proper to cure or circumscribe*, the use of Mr. Baynton's admirable adhesive strap. Mr. Cross complains, that the French spread the sticking-plaster very "clumsily," and apply it "aukwardly." Cold applications are seldom or never used in local inflammations. The following remark of our English observer is alike correct and judicious. We must conclude with it.

"From the hospital practice of the Parisian surgeons as well as physicians, one would suppose that the humoral pathology is still held in great credit amongst them; for to order a seton or issue is as common, as with us to direct keeping open the bowels. Their list of diseases which it is dangerous to cure, has been extended to an extravagant length: yet, although not at all disposed to fall in with all their opinions, I was pleased and instructed by seeing them look so much to the prevention of disease, and by observing that they did not always take the quickest means of removing present symptoms, without considering what might be the consequences, or what the diseases that might possibly arise."

Of the general character and merits of Mr. Cross's "Sketches," we have before spoken; and, after an examination thus long and rigorous, we find no reason to alter the opinion then delivered. We regret that he has been so lavish of French phraseology. Its frequent introduction must be productive of great vexation and embarrassment to the unlearned reader, and gives to his work an air of flippancy and affectation — vices from the pollution of which the book, in other respects, is singularly exempt. Had its employment been restricted to those cases in which the English language offered not equal facilities of expression, all very well! But out of the numerous terms and sentences borrowed from the French, there are few, very few, which might not have been delivered, with equal clearness and precision, in the "vulgar tongue."

